# Exercise and cancer: from “healthy” to “therapeutic”?

**DOI:** 10.1007/s00262-017-1985-z

**Published:** 2017-03-21

**Authors:** Manja Idorn, Per thor Straten

**Affiliations:** 1 Department of Hematology, Center for Cancer Immune Therapy (CCIT), University Hospital Herlev, Herlev Ringvej 75, 2730 Herlev, Denmark; 2 Department of Immunology and Microbiology, University of Copenhagen, Copenhagen, Denmark

**Keywords:** Exercise, NK cells, Immune cell mobilization, Tumor infiltration, Combination treatment, CIMT 2016

## Abstract

Exercise improves functional capacity and patient-reported outcomes across a range of cancer diagnoses. The mechanisms behind this protection have been largely unknown, but exercise-mediated changes in body composition, sex hormone levels, systemic inflammation, and immune cell function have been suggested to play a role. We recently demonstrated that voluntary exercise leads to an influx of immune cells in tumors, and a more than 60% reduction in tumor incidence and growth across several mouse models. Given the common mechanisms of immune cell mobilization in mouse and man during exercise, we hypothesize that this link between exercise and the immune system can be exploited in cancer therapy in particular in combination with immunotherapy. Thus, we believe that exercise may not just be “healthy” but may in fact be therapeutic.

Physical exercise has documented impact on health and well-being of humans, and is associated with a reduced risk of mortality [1]. Exercise has also been shown to have a preventive effect on the risk of cancer, i.e., leisure-time exercise is associated with a lower cancer incidence. Although epidemiological studies of exercise and its impact on slowly developing diseases are often self-reported and might be biased by other lifestyle factors and often, available data strongly suggest exercise to be preventive for cancer [2, 3]. Concerning a potential role for exercise in cancer prognosis and therapy, physical activity has been reported to be associated with a lowered risk of disease recurrence (breast cancer and colorectal cancer) [3, 4]. However, data are needed in other cancers, and not much is known in terms of the optimal timing and type of activity. Moreover, the mechanism by which exercise impact on cancer risk and prognosis is largely unknown.

We recently took advantage of several murine tumor models to study the impact of exercise on cancer, and could demonstrate that voluntary exercise (wheel running) leads to a significant reduction in tumor size or incidence across the applied models [5]. Thus, exercise had a significant impact on tumor size in the transplanted lewis lung cancer and B16 melanoma models—the latter studied both as subcutaneous (s.c.) tumors, as well as lung metastases established upon intravenous administration of cancer cells. Moreover, we could show an impact of exercise on tumor size and incidence on liver cancer using a diethylnitrosamine (DEN) induced liver cancer model, as well as the spontaneous melanoma model GrM1. To gain insight into the mechanism, we used the transplanted B16 melanoma model and compared tumors from exercise and control animals and, by microarray, showed that pathways associated with immune function were significantly up-regulated in tumors from exercise animals. Using real-time PCR, we validated the elevated expression levels of selected transcripts, and took advantage of immunohistochemistry (IHC) and flow cytometry, to substantiate these findings at the cellular level. Thus, tumors from exercise animals had a much denser infiltration of immune cells; T cells, B cells, dendritic cells, and NK cells. Since in particular NK cells were repeatedly increased in both subcutaneous and lung metastases of B16 tumors, we first used nude mice to see if exercise would still have an impact in the absence of T and B cells. Indeed, this was the case. As a next step, we went back to using B16 melanoma in wild type C57BL/6 mice, and used anti-asialo monoclonal antibody to clear NK cells from the animals. Strikingly, this completely abolished the effect of exercise, demonstrating that at least in the used transplanted tumor model, NK cells were necessary and sufficient for the effect [5]. NK cells are known to be capable of killing cancer cells [6], but a link between NK cells and the exercise associated anti-tumor response had not previously been scrutinized.

Exercise in humans is associated with a range of physiological changes, the magnitude of which is influenced by the intensity and duration of the exercise. Thus, there is an increase in cardiac output to meet oxygen demands, and a dramatic change in the pattern of blood flow. The metabolic rate goes up, and glucose consumption as well as output is increased, as is lactate levels due to anaerobic metabolism in muscle cells. Moreover, the endocrine system plays a key role in integrating the physiological responses both during rest and exercise [7]. To this end, catecholamines including epinephrine (adrenaline), and norepinephrine (noradrenaline) are significantly elevated during exercise through increased release from the adrenal glands. These hormones are part of the fight-or-flight response associated with increases in heart rate, blood pressure, blood glucose levels—and; immune function. To the latter, although there are conflicting data on the subject, there is consensus that acute exercise leads to a rapid increase in blood counts of various immune cells [7], followed by a drop to below baseline in turn followed by normalization of cell counts [8]. The most sensitive immune cell type to acute exercise is the NK cell [9], which are mobilized within minutes of exercise [10]. Maximal mobilization of NK cells is achieved after 30 min of exercise, and prolonged training does not lead to increased NK cell levels, but max level of NK cells can be maintained up to 3 h by continued training [9]. NK cells were initially characterized by being able to kill target cells without prior priming [6], and they normally function to clear virus infected, stressed, or transduced cells. This exercise-induced mobilization of NK cells is supposedly installed mainly by an increase in catecholamines. To this point, among leucocytes, NK cells express the highest levels of β-adrenergic receptors which is the receptor for catecholamines, especially norepinephrine and epinephrine [11], and the exercise associated mobilization of NK cells can be mimicked by administration of epinephrine [12].

In part based on the above, we first looked at serum levels of epinephrine and norepinephrine in exercise and control animals, and found that exercise animals had significantly higher amounts of both catecholamines. Moreover, we could block the effect of exercise on the increase in NK cell mobilization, immune cell influx to tumors, as well as impact on tumor progression by administration of the β-blocker propranolol [5]. Aiming at scrutinizing if epinephrine was alone responsible, we administered epinephrine to the animals, which did, indeed, impact on NK frequencies, immune cell infiltration into tumors, and size of tumors—but not as pronounced an effect as exercise. Thus, epinephrine is a key molecule in the exercise associated improved tumor control by cells of the immune system.

Since administration of epinephrine only partially mimicked exercise, we considered other molecules that could potentially play a role.

Exercise is associated with the release of myokines from the contracting muscles. One of the key myokines is interleukine-6 (IL-6) which is also known from its wide ranging and sometimes contrasting effects within the immune system. IL-6 is described to be pro-inflammatory in settings of bacterial infections and chronic inflammation, yet act anti-inflammatory by inhibiting TNF-α and IL-1, and activation of immune suppressive IL-10 [13]. During exercise, the plasma level of IL-6 increases rapidly in an intensity-dependent manner, through the release from exercise-engaged muscles [14]. As NK cells express the IL-6 receptor complex, we speculated whether exercise-mediated IL-6 release could play a role in, i.e., add to the effect of administration of epinephrine. We first cleared IL-6 using an IL-6 specific monoclonal antibody (mAb) which led to a diminished tumor control and also a less pronounced immune cell infiltration to the tumor; however, we could not mimic the role of IL-6 by administration of recombinant cytokine. Certainly, there could be many reasons for this: dosing, route of administration, timing related to levels of epinephrine, etc. At least one role IL-6 could play would be to increase mobilization of NK cells, since NK cells leaving the thymus were selectively expressing the IL-6α receptor. Cancer cells—including melanomas—quite often express high levels of IL-6, which could, in turn, play a role in homing of NK cells expressing the IL-6 receptor. However, this is purely speculative and more work is needed to clarify the role of IL-6 in NK cell homing to the tumor site.

The data summarized above could have several important implications in humans both in relation to cancer incidence and therapy.

First, human NK cells express β-adrenergic receptors and as already mentioned administration of epinephrine leads to mobilization of NK cells. Similarly, bouts of acute exercise lead to mobilization of leucocytes most pronouncedly NK cells, and thus, increase in systemic NK cell frequencies is a key immunological feature of exercise in man as well as mouse [15]. It should be mentioned, however, that in all sc. B16 tumors from exercise mice, we also saw increased numbers of T cells. T cells also express β-adrenergic receptors and are thus also mobilized by epinephrine. Moreover, killing of cancer cells by NK cells may have led to uptake of tumor antigen by dendritic cells, and induction of tumor-specific T-cell responses. In terms of the function of NK cells, although different in terms of cell surface markers and signalling molecules [16], there are basic similarities in terms of being similarly capable of recognizing and killing of cancer cells as a functional response to recognizing “missing self” [6]. NK cells may also recognize cancer cells by activating receptors, e.g., NKG2D recognizing ligands expressed on cancer cells [17]. Thus, exercise in humans mobilizes NK cells, and human NK cells are efficient cancer cell killers—and, therefore, could play a protective or therapeutic role in cancer. To this end, Moore and colleagues recently pooled data from 12 prospective cohorts with self-reported physical activity for association with incidence of 26 types of cancer, and could demonstrate that leisure-time physical activity is significantly associated with a lower risk of cancer, e.g., cancers of the oesophagus, liver, lung, kidney, colon, breast, and as well as leukemia and myeloma [2]. Strikingly, two cancers showed physical activity associated with higher risk of cancer, melanoma, and prostate cancer. For melanoma, this could supposedly be due to sun exposure during exercise. For advanced prostate cancer, there was no relationship between physical activity and cancer, suggesting that men that exercise, are more prone to be examined and diagnosed—in some cases with indolent prostate cancer. Nonetheless, with two exceptions, physical activity is associated with lower risks of a range of cancer types. Obviously, these data do not by itself reveal insight into the molecular or cellular background but underscore that the exercise associated immune mobilization could potentially play a role in cancer prevention. To this end, a key feature of the protective role of exercise in our mouse studies was an increased number of immune cells in tumors from exercise animals [5], a feature known to be associated with longer survival in humans [18]. Moreover, immune infiltration can be studied in humans by serial biopsies before and after exercise. Thorough studies of the composition of immune cells infiltrating the tumor could possibly reveal data to support the notion of improved immunological control of tumor progression, and set the stage for combination treatment as given in more detail below.

Second, in our fast growing transplantable B16 melanoma model, NK cells were responsible for the anti-cancer effect of exercise, and NK cells had significant effect in the absence of T and B cells. As a consequence, it is possible that fast growing transplantable models in which NK cells do not play a substantial role would see limited or no effect of exercise. In our B16 model, the most pronounced effect was achieved when exercise was initiated prior to inoculation of cancer cells. We also did studies in which exercise was initiated concurrent with cancer cell inoculation and saw only a trend towards smaller tumors in the exercise group [5]. Hence, it makes sense to speculate that prior exercise implies that more NK cells are in circulation and ready to home to the inoculation site and kill cancer cells immediately at the site of injection. Thus, we have no evidence that NK cells alone are responsible for the effect using more clinically relevant tumor models. In more slowly developing tumor models, e.g., the DEN or the GrM1 model, we are currently following the hypothesis that NK cells are key cells in delivering the “spark” that could be a main denominator for setting the stage for induction of a full scale response involving all appropriate cell types necessary for an anti-cancer immune attack: most importantly dendritic cells and T cells. This would be in line with the data suggesting NK cells to play a main role in very early phases of tumorigenesis and a less pronounced role once the tumor is established [18, 19]. This notion is supported by the fact that solid tumors comprise quite few NK cells, whereas infiltrates of T cells are often present in more substantial numbers [20]. A key role of NK cells in protection rather than therapy is also suggested from a study in which NK cell levels were studied longitudinally with an 11 year interval using a functional cytotoxicity readout, which showed that a high level of cytotoxic activity among peripheral blood lymphocytes was associated with a lower risk of cancer [21]. Again, this suggests NK cells as active in early tumor surveillance.

In terms of translation to a human setting, maybe, the most important finding of our recent study is the impact on exercise shown using the chemically induced model (DEN) or the spontaneous melanoma model. In the DEN model, we saw a decreased in tumor incidence going from 70 to 30%, and given the fact that the immune infiltrate in B16 showed a significant increase not only in NK cells, but also dendritic cells, B cells, and T cells, we hypothesize that in the slow growing more clinically relevant models, NK cells may be involved with early surveillance, while other immune subsets play a more important role in anti-tumor responses against the established tumor.

Third, given the immune cell mobilizing and tumor infiltrating effect of exercise, this could potentially play a role as combination or conditioning partner to immune therapy. To this end, the immune check point inhibitory mAb are now approved in several cancers and the list of cancer indications is certain to increase. In particular, blockade of the PD-1 axis by mAbs seems to induce clinical responses by unleash of spontaneous tumor-specific immune responses at the tumor site [22]. Importantly, data are accumulating to suggest that patients whose tumors are characterized by a brisk infiltration of immune cells are more prone to respond to treatment [23]. As consequence, huge research efforts are undertaken to scrutinize methods by which tumors with limited or absent immune cell infiltrates, i.e., “cold tumors” can be turned into “hot” tumors with a brisk infiltration of anti-tumor immune cells, T cells, NK cells, dendritic cells, etc. Therefore, exercise prior to PD-1 therapy could represent a tool that condition patients to immunotherapy by increasing the immune infiltrate in the tumor, and in turn increase the chance for clinical response. Adoptive cell transfer (ACT) using tumor infiltrating lymphocytes (TIL) represents another breakthrough in immunotherapy of cancer. A very obvious requirement for clinical success is the presence of tumor-specific T cells in the tumors, since these cells prepare the platform for expansion in vitro and administration of high numbers of tumor-specific T cells back to the patient. In melanoma patients, 50% of patients respond to treatment with an impressive 20% experiencing lasting complete, i.e., supposedly curative responses [24, 25]. As mentioned, exercise prior to surgery for harvest of T cells could possibly promote the quantity of cells and maybe also the quality of “new” immune cells in tumor. Prior to clinical testing, this could be studied by harvesting of serial biopsies for studies of immune cell infiltration and in vitro functionality of immune cells, in the pre-clinical and clinical setting, by studying needle biopsies before and after monitored exercise programs. Several lines of evidence point at the immune system as being crucial also for the efficacy of chemotherapy [26]. If substantiated by human studies, exercise could play a role by improving also response to the conventional treatments [27].

Cancer immunotherapy is now clinically validated in many cancers, and even in very late stage patients, lasting complete responses are not uncommon. However, for most treatments, the majority of patients do not respond and predictive markers are missing. Hence, there is an urgent call for characterization of predictive markers and tools to increase response to therapy. To the latter, exercise is known to be healthy in an array of aspects in the life of modern human beings. Based on our data in mouse models, we suggest that exercise could represent a suitable combination partner to immune therapy in cancer patients, facilitating improved response rates and more frequent complete lasting responses. Moreover, exercise may directly—as a key component of a healthy lifestyle—delay or prevent tumor development.

## Abbreviations

DEN: Diethylnitrosamine
IHC: Immunehistochemistry
IL-6: Interleukine-6
mAb: Monoclonal antibody
NK cell: Natural killer cell
s.c.: Subcutaneous
TNF-α: Tumor necrosis factor alpha
